# Improving power of genome-wide association studies via transforming ordinal phenotypes into continuous phenotypes

**DOI:** 10.3389/fpls.2023.1247181

**Published:** 2023-11-02

**Authors:** Ming Yang, Yangjun Wen, Jinchang Zheng, Jin Zhang, Tuanjie Zhao, Jianying Feng

**Affiliations:** ^1^ Key Laboratory of Biology and Genetics Improvement of Soybean, Ministry of Agriculture/Zhongshan Biological Breeding Laboratory (ZSBBL)/National Innovation Platform for Soybean Breeding and Industry-Education Integration/State Key Laboratory of Crop Genetics & Germplasm Enhancement and Utilization/College of Agriculture, Nanjing Agricultural University, Nanjing, China; ^2^ College of Science, Nanjing Agricultural University, Nanjing, China

**Keywords:** ordinal trait, genome-wide association study, salt-alkali tolerance, soybean, hierarchical data

## Abstract

**Introduction:**

Ordinal traits are important complex traits in crops, while genome-wide association study (GWAS) is a widely-used method in their gene mining. Presently, GWAS of continuous quantitative traits (C-GWAS) and single-locus association analysis method of ordinal traits are the main methods used for ordinal traits. However, the detection power of these two methods is low.

**Methods:**

To address this issue, we proposed a new method, named MTOTC, in which hierarchical data of ordinal traits are transformed into continuous phenotypic data (CPData).

**Results:**

Then, FASTmrMLM, one C-GWAS method, was used to conduct GWAS for CPData. The results from the simulation studies showed that, MTOTC+FASTmrMLM for ordinal traits was better than the classical methods when there were four and fewer hierarchical levels. In addition, when MTOTC was combined with FASTmrEMMA, mrMLM, ISIS EM-BLASSO, pLARmEB, and pKWmEB, relatively high power and low false positive rate in QTN detection were observed as well. Subsequently, MTOTC was applied to analyze the hierarchical data of soybean salt-alkali tolerance. It was revealed that more significant QTNs were detected when MTOTC was combined with any of the above six C-GWAs.

**Discussion:**

Accordingly, the new method increases the choices of the GWAS methods for ordinal traits and helps to mine the genes for ordinal traits in resource populations.

## 1 Introduction

The hierarchical data (HData), phenotypic data for ordinal traits, is commonly used to describe many important traits in crop germplasm resources. This includes count data for quantitative traits and hierarchical data for resistance traits, such as the number of main stem nodes (Chang et al., 2018), the number of branches (Shim et al., 2019), and disease resistance (Megerssa et al., 2020). Ordinal traits are important in crop breeding and have a considerable impact on crop yield and quality. Genome-wide association studies (GWAS) for ordinal traits can further promote the mining of relevant excellent genes, which plays a key role in molecular design breeding and gene cloning. Cuevas et al. (2018) divided the degree of infection of anthracnose-inoculated sorghum leaves into five levels and identified three loci for anthracnose resistance in chromosome 5 using the GWAS methods. Chang et al. (2018) detected three loci significantly associated with “the number of nodes on the main stem” in 368 soybean cultivars with 62,423 SNPs. Meanwhile, Shim et al. (2019) identified five quantitative trait nucleotides (QTNs) for soybean branch number via GWAS and linkage analysis and mined a candidate gene *Glyma.06g210600*.

Ordinal traits are discrete traits that are controlled by multiple genes. However, their phenotypic data is hierarchical and non-continuous and contains relatively limited information; accordingly, GWAS for ordinal traits is more complex than that for continuous quantitative traits. The threshold model represents a reasonable method for the genetic analysis of ordinal traits, and most association mapping methods are developed under this framework (Xu et al., 2005; Osval et al., 2015). Generalized linear model is based on the threshold model and link phenotypic data with latent variables through a link function. They are widely used for genetic analysis of ordinal traits and can deal with non-normal data (Feng et al., 2013; Song et al., 2016; Wang et al., 2018). The logistic regression model is another classical way for dealing with association studies of ordinal traits (Tan et al., 2007; Hoggart et al., 2008; Wu et al., 2009; Jiang et al., 2021). When sample size is limited, the application of a set-valued (SV) system model can improve the statistical power and the accuracy of parameter estimation (Bi et al., 2015). Bayesian and maximum likelihood methods are both widely used for parameter estimation in GWAS (Xu et al., 2005; Hoggart et al., 2008; Wang et al., 2018), while several studies have also employed non-parametric methods for association analysis of ordinal traits (Sun et al., 2016; Wang et al., 2017; He and Kulminski, 2020). However, most of them were either single-locus or were only suitable for the analysis of binary traits, and they had very few applications in crop. GWAS for continuous quantitative traits and single-locus methods are currently the main methods used for association analysis of ordinal traits; however, both have low power in QTN detection.

Accordingly, in this study, we proposed a method for transforming ordinal phenotypes into continuous phenotypes (MTOTC). First, the hierarchical phenotypic data for ordinal traits (HData) was transformed into continuous phenotypic data (CPData). Subsequently, FASTmrMLM (Tamba and Zhang, 2018), one GWAS method suitable for continuous quantitative traits, was used to perform GWAS for CPData. In Monte Carlo simulation studies, we validated the feasibility of the new method through the statistical power, false-positive rate in QTN detection and the accuracies for the estimates of QTN effects and positions, and obtained the number of hierarchical levels suitable for MTOTC+FASTmrMLM. The new method was validated by re-analyzing the salt-alkali resistance traits in soybean germplasm resource population of Zhang et al. (2014) and Zhou et al. (2015). This study provides more choices for association analysis of ordinal traits and helps to identify excellent genes for important complex traits in crops.

## 2 Theory and methods

Here we proposed a method, named MTOTC, to transform the discrete hierarchical data (HData) of ordinal traits into continuous phenotypic data. Then, GWAS for continuous quantitative traits (C-GWAS) are used to analyze the transformed continuous phenotypic data. The new method was described as below.

### 2.1 Genetic mapping population

In Monte Carlo simulation studies, 199 *Arabidopsis thaliana* lines harboring 10,000 SNPs with a minimum allele frequency >0.1 (Atwell et al., 2010) were selected as the genetic mapping population. For real data analysis, the population was comprised of 286 soybean cultivars assessed for salt-alkali tolerance, the phenotypic data consisted of the main root length index in 2009 and 2010 (Zhang et al., 2014), and the marker data were 54,296 high-quality SNP markers present in Zhou et al. (2015).

### 2.2 Method for transforming ordinal phenotypes into continuous phenotypes

**Figure 1:**
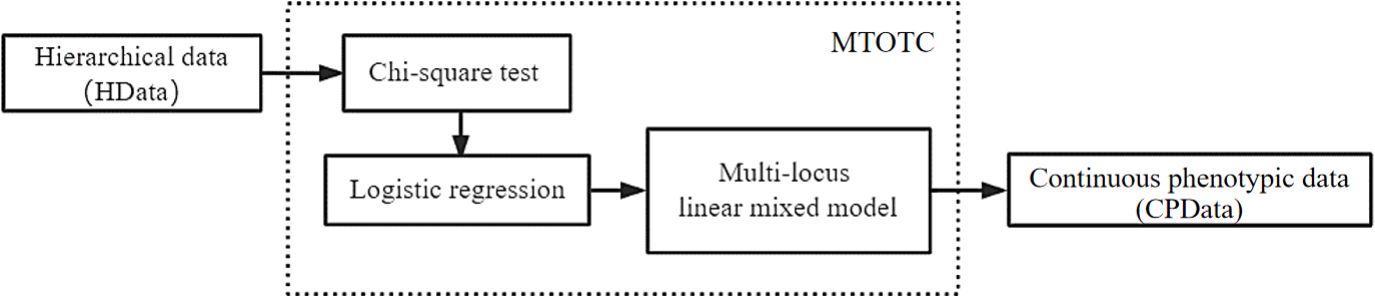
Technology framework of the MTOTC method in this study.

To transform ordinal phenotypes into continuous phenotypes, we proposed the MTOTC method. In detail, the Chi-square test and logistic regression were used to initially select the SNPs that were significantly related to the trait. Subsequently, these significant SNPs and ordinal phenotypes were used to construct a multi-locus model, Bayesian method was used to estimate the SNP effects, and the effect estimates were used to predict the continuous phenotypic data (CPData). This is MTOTC. Then, the predicted CPData is analyzed by C-GWAS methods, such as FASTmrMLM (**Figure 1**).

#### 2.2.1 The Chi-square test and logistic regression

The Chi-square test in R 4.0.5 (function “chisq.test”) was used to scan the SNPs in the whole genome using a single marker method (*P*-value ≤0.05). To further improve the quality of the significant correlated SNPs in the initial screening for reducing interference and improving detection accuracy, logistic regression was used as a secondary SNP screening method. Logistic regression was performed using function “glm” (2 hierarchical levels) and “polr” (the number of hierarchical levels greater than 2) with a *P*-value ≤0.05. The aim of this step was to further eliminate SNPs that were not associated with the traits for simplifying the iterations in the following multi-locus genetic model.

#### 2.2.2 Multi-locus genetic model

Based on the potentially associated markers identified in the above-described initial screening, a multi-locus model was established to transform ordinal phenotypes into continuous phenotypes. The linear model is expressed as:


(1) $$y=W\alpha +\sum_{i=1}^{q}X_{i}\beta_{i}+\epsilon$$

where *y* represents n×1 ordinal phenotype vector, with *n* representing sample size; $W=\left(w_{1},\;w_{2},\;\ldots ,\;w_{c}\right)\;$ represents n×c matrix of covariates (fixed effects), including a column vector of **1** and population structure, and represents c×1 vector of fixed effects, including intercept; $X_{i}$ and represent respectively n×1 genotype vector and effect of the *i-*th potential associated SNP; *q* represents the number of SNPs selected in the initial screening step; $\epsilon \sim {\text{MVN}}_{n}\left(0,\;\sigma_{e}^{2}{\text{I}}_{n}\right)\;$ represents n×1 error vector.

The population structure **Q** matrix used in the linear model was calculated using Structure software (Pritchard et al., 2000). Based on the **Q** matrix, the population is divided into corresponding subgroups, and the optimal subgroup number **K** value is determined according to the corresponding standard, yielding the final **Q** matrix. The optimal value of the *Arabidopsis* population structure was calculated as **K**=2, and the optimal value of the salt-alkali tolerant soybean population structure in the actual study was **K**=3.

#### 2.2.3 Parameter estimation

In the second step of the novel method, a multi-locus linear mixed model for transforming ordinal phenotypes into continuous phenotypes was established, based on the empirical Bayesian algorithm (Xu, 2010). And significant loci were screened in threshold value LOD=3.0.

In model (1), set $\beta_{i}$ to obey the following prior normal distribution:


$$P\left(\beta_{i}|\sigma_{i}^{2}\right)=N\left(0|\sigma_{i}^{2}\right)$$

$$P\left(\sigma_{i}^{2}|\tau ,\;\omega \right)\propto {\left(\sigma_{i}^{2}\right)}^{-\frac{1}{2}\left(\tau +2\right)}\times exp\left(-\frac{\omega }{2\sigma_{i}^{2}}\right)$$

The parameters were estimated using empirical Bayes, as follows, and the Newton–Raphson method.


$$\sigma_{i}^{2}=\frac{E\left(\beta_{i}^{T}\beta_{i}\right)+\text{ω}}{\tau +3}$$

$$\alpha ={\left(W^{T}V^{-1}W\right)}^{-}W^{T}V^{-1}\text{y}$$

$$\sigma_{e}^{2}=\frac{1}{n}{\left(y-W\alpha \right)}^{T}\left(y-W\alpha -\sum_{i=1}^{q}X_{i}E\left(\beta_{i}\right)\right)$$

$$E\left(\beta_{i}\right)=\sigma_{i}^{2}X_{i}^{T}V^{-1}\left(y-W\alpha \right)$$

Among them,


$$E\left(\beta_{i}^{T}\beta_{i}\right)=E\left(\beta_{i}^{T}\right)E\left(\beta_{i}\right)+tr\left[Var\left(\beta_{i}\right)\right]$$

$$Var\left(\beta_{i}\right)=I\sigma_{i}^{2}-\sigma_{i}^{2}X_{i}^{T}V^{-1}X_{i}\sigma_{i}^{2}$$

(τ, ω)=(0, 0)

$$V=\sum_{i=1}^{q}X_{i}X_{i}^{T}\sigma_{i}^{2}+\text{I}\sigma_{e}^{2}$$

Then, the empirical Bayesian estimates of these SNPs effects were obtained in the multi-locus model (1) based on the selected significant SNP markers and ordinal phenotype, and estimates of these effect were used to predict the phenotype, obtaining the continuous phenotypic data (CPData) of ordinal trait.

### 2.3 GWAS with MTOTC method for ordinal trait

When continuous phenotypic data was obtained by the above MTOTC method, a C-GWAS method could be used to detect significant loci. In this work, FASTmrMLM, one C-GWAS method, was used. So loci significantly associated with ordinal traits were detected by FASTmrMLM using the obtained continuous phenotypic data and the potential associated markers identified in the above-described initial screening. The GWAS method is henceforth referred to as MTOTC+FASTmrMLM. Moreover, the effects of five other C-GWAS (FASTmrEMMA, mrMLM, ISIS EM-BLASSO, pLARmEB, and pKWmEB) methods are also discussed based on the MTOTC method for ordinal trait, in order to verify the feasibility of MTOTC.

### 2.4 Monte Carlo simulation datasets for ordinal trait

We conducted six simulation studies to evaluate the feasibility of the new method. For each study, the loci 278, 2143, 2054, 3698, 1716, 6178, and 8501, located on chromosomes 1, 2, 2, 2, 1, 4, and 5, respectively, were selected as the causal loci related to the simulated trait. There were three types of phenotypic data in the simulation experiment—original data (OData), which were continuous and generated by Monte Carlo simulation; HData, which were generated from the above OData according to specific distribution proportions (i.e., classification proportion of phenotype distribution); and CPData, which were generated from the above HData by MTOTC. Then, FASTmrMLM, one multi-locus C-GWAS algorithm, was used to conduct GWAS for CPData.

## 3 Results

### 3.1 Monte Carlo simulation studies

#### 3.1.1 Threshold value in the initial screening

To determine the most suitable threshold value for the Chi-square test and logistic regression in the initial screening, four probability thresholds (0.0001 [i.e., 1/SNP number], 0.01, 0.05, and 0.10) were set for the Chi-square test in the first simulation study, while three probability thresholds (0.0001 [i.e., 1/SNP number], 0.01, and 0.05) were set for logistic regression. The Chi-square test can eliminate a large number of SNPs that are not significantly related to a given phenotype. However, the simulation study showed that some SNPs screened in the above Chi-square test (those with a *P*-value >0.98 and an unusually large absolute value of effect estimate in logistic regression) were not truly related to the phenotype and interfered greatly with subsequent association analysis. Therefore, to further improve the quality of the screened significantly related SNPs and detection accuracy, logistic regression was used as a secondary screening method for SNPs in MTOTC.

**Figure 2:**
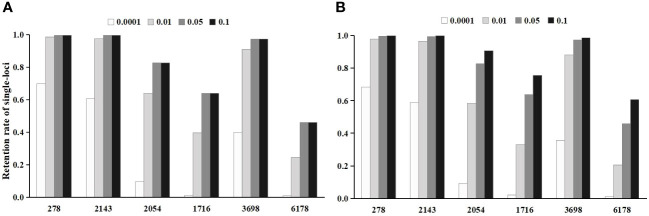
The effect of threshold value on the single-locus retention rate after the initial screening. **(A)** is the single-locus retention rate after chi-square test screening; **(B)** is the single-locus retention rate after logistic regression screening.

**Figure 3:**
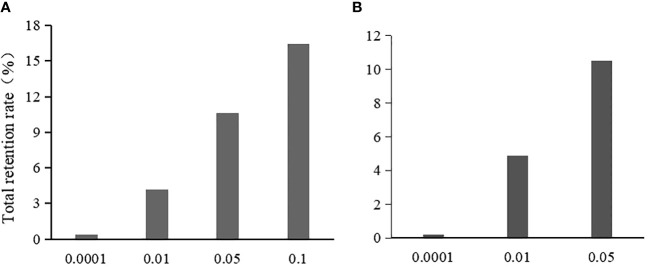
The effect of threshold value on the total retention rate after the initial screening. **(A)** is the total retention rate in chi-square test; **(B)** is the total retention rate in logistic regression.

In the Chi-square test, the single-locus retention rate decreased with decreasing *P*-values (i.e., threshold values) (**Figure 2A**). For instance, the single-locus retention rate at loci 278 and 2143 with *P*-values of 0.05 and 0.10 was as high as 96.62%~99.68%, which are very close. When the *P*-value was 0.01, the single-locus retention rate began to decrease, and when the *P*-value was 0.0001, the retention rate dropped to between 59.06% and 68.45%. Moreover, the total retention rate (i.e., the proportion of retained loci among the total loci after chi-square test screening) was the lowest when the *P*-value was 0.0001, followed by 0.01, 0.05, and 0.10 (**Figure 3A**).

In logistic regression after the Chi-square test, the single-locus retention rate was the highest when the *P*-value was 0.05 (**Figure 2B**). For instance, the retention rates of loci 278 and 2143 were as high as 97.56%~99.68% when the *P*-value was 0.01 or 0.05; when the *P*-value was 0.0001, the retention rate dropped to between 60.51% and 69.88%. Additionally, the total retention rate was the lowest (only 0.22%) when the *P*-value was 0.0001, followed by 0.01 and 0.05 (**Figure 3B**).

Owing to too low single-locus retention rate at the *P*-values of 0.01 and 0.0001, the two *P*-values were unsuitable as a threshold for initial screening. Although the total retention rate was high when the *P*-value was 0.10, this *P*-value retains more loci that are not associated with the trait, in which it did not contribute to simplifying the model. Therefore, the probability threshold *P*=0.05, which is commonly used in statistics, was selected as the probability threshold for the Chi-square test and logistic regression of the initial screening in this study. In addition, we also investigated the effect of threshold value on the single-locus retention rate and the total retention rate under different proportions distribution in binary data and the similar results were observed.

#### 3.1.2 MTOTC+FASTmrMLM displayed greater power than other classical mapping methods

In Monte Carlo simulation studies, the GWAS results of hierarchical data using MTOTC+ FASTmrMLM were compared with those using two classical mapping methods (Chi-square test and logistics regression) (**Table 1**). The results showed that these methods had greater power at the three loci 278, 2143, and 3698, but had less power (<10%) at the other four loci. Compared with the two classical mapping methods, MTOTC+FASTmrMLM had higher power at the three loci 278, 2143, and 3698, and lower false-positive rate, when the number of hierarchical levels of HData was ≤4. The power of the classical methods was higher in a few instances, it was less than 1.5-fold that of MTOTC+FASTmrMLM, but their false-positive rates were 6.8–9.5-fold higher than that of MTOTC+FASTmrMLM. In addition, the results showed that when the number of hierarchical levels was<5, MTOTC+FASTmrMLM was more suitable for HData analysis as compared with FASTmrMLM alone. Moreover, in **Table 1**, MTOTC+FASTmrMLM had a relatively higher F1 score, especially for binary data (HData with two hierarchical levels). Here the F1 score combines the precision and recall, it is used to effectively measure the accuracy of the statistical methods and balance power and FPR. Therefore, MTOTC is recommended for the analysis of HData under four or fewer hierarchical levels.

**Table 1:** Comparison of different genome-wide association study methods.

Hierarchical number	Locus		Chi-square test	logistic regression	FASTmrMLM	MTOTC+ FASTmrMLM
2	Power(%)	278	66.20	22.50	41.85	57.38
		2143	57.70	19.40	28.62	55.98
		3698	18.40	10.10	9.89	22.76
	Mean of Power (%)		20.87	7.60	13.84	19.54
	FPR (‰)		7.27	0.07	0.44	0.77
	F1 score		0.04	0.13	0.16	0.17
3	Power(%)	278	62.00	71.30	53.41	70.87
		2143	56.40	57.20	45.76	66.64
		3698	20.00	26.90	19.66	36.82
	Mean of Power (%)		20.47	23.30	22.06	26.53
	FPR (‰)		6.15	4.77	0.45	0.70
	F1 score		0.04	0.06	0.24	0.24
4	Power(%)	278	68.40	80.30	65.70	75.98
		2143	58.50	65.40	58.71	71.83
		3698	20.80	37.30	27.76	45.69
	Mean of Power (%)		21.77	27.37	28.29	28.53
	FPR (‰)		8.11	5.90	0.45	0.63
	F1 score		0.03	0.06	0.29	0.26

#### 3.1.3 The effect of the number of hierarchical levels on the new method

The third simulation study investigated the effect of the number of hierarchical levels on MTOTC. Based on symmetrical distribution, the number of hierarchical levels was set to 2, 3, 4, and 5, respectively, and the number of replicates was 10,000. Meanwhile, we compared the results of OData, HData and CPData using FASTmrMLM.

**Figure 4:**
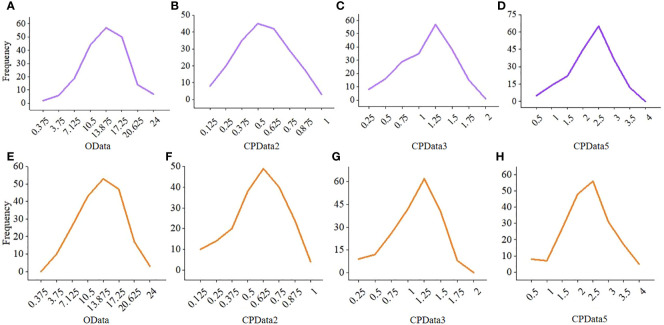
The frequency distribution of the OData and the corresponding CPData for different hierarchical levels in the 10th and 613th repetition. **(A–D)** is the 10th repetition, **(E–H)** is the 613th repetition. CPData2 transformed from HData of two hierarchical levels by MTOTC; CPData3 transformed from HData of three hierarchical levels by MTOTC; CPData5 transformed from HData of five hierarchical levels by MTOTC.

Compared with CPData from the other hierarchical levels, the distribution of CPData2 (i.e., the CPData converted from the HData of 2 hierarchical levels by MTOTC) was closer to the original data (OData). First, the frequency distribution of the CPData was closer to that of the OData when the hierarchical level was low, especially when it was equal to 2 (**Figure 4**). As the number of hierarchical levels increased, the peak of CPData began to shift to the right and was far from the peak of the OData, which was expected to affect the GWAS results. The frequency distribution of the OData and the corresponding CPData with different hierarchical levels in the 10th and 613th replicates, randomly selected out of the 10,000 replicates using the uniformly distributed random number generator in R, is shown in **Figure 4**. Second, the range of the coefficient of variation (*CV*) of the OData was between 29.5% and 55.5%. Among the 10,000 replicates, the number of replicates beyond the *CV* range of the OData (4.09%, 18.94%, 21.47%, and 25.37% of CPData2, CPData3, CPData4, and CPData5, respectively) also increased with increasing hierarchical level. Thus, the *CV* range of CPData2 was the closest to that of the OData. Third, among the 10,000 replicates, the skewness range between the CPData and the OData was the closest at 2 hierarchical levels. Among them, the skewness range of the OData was between −1.00 and 0.46 and the range of CPData2 was between −1.28 and 0.35. As the number of hierarchical levels increased, the skewness of the CPData gradually deviated from that of the OData; the kurtosis showed the same tendency as the skewness.

MTOTC performed well for the estimates of QTN position under different numbers of hierarchical levels. The position estimates via MTOTC+FASTmrMLM (i.e., the position estimates of the CPData via FASTmrMLM) were unbiased at loci 278, 2143, and 3698 (**Supplementary Table 1**). Although the position estimates at loci 2054 and 8501 in CPData2, and at loci 1716 and 6178 in all the CPData were biased, the relative mean absolute deviations of their position estimates were all less than 8.96E-05. The accuracy of the estimates of QTN positions for ordinal traits was significantly improved by MTOTC when the number of hierarchical levels was less than 5, i.e., the estimates of QTN positions for the CPData were better than those for the HData when FASTmrMLM was used (**Supplementary Table 1**).

The effect of MTOTC on the relative power at loci 278, 2143, and 3698 was the greatest when the number of hierarchical levels is equal to 2 (**Supplementary Figure 1**). Here, “the effect of MTOTC on the relative power” refers to the increment of the relative power of CPData compared to the relative power of HData. The relative power of the CPData (50%~100%) was significantly higher than that of the HData (22%~88%) and was relatively closer to the power of the OData. When the number of the hierarchical levels of the CPData was less than or equal to 5, the relative power exhibited an increasing trend with increasing the number of hierarchical levels and was significantly superior to that of the HData.

The false-positive rates of CPData2, CPData3, CPData4, and CPData5 via MTOTC+FASTmrMLM were 0.77‰, 0.70‰, 0.63‰, and 0.55‰, respectively.

#### 3.1.4 The effect of the number of replicates on the new method

**Figure 5:**
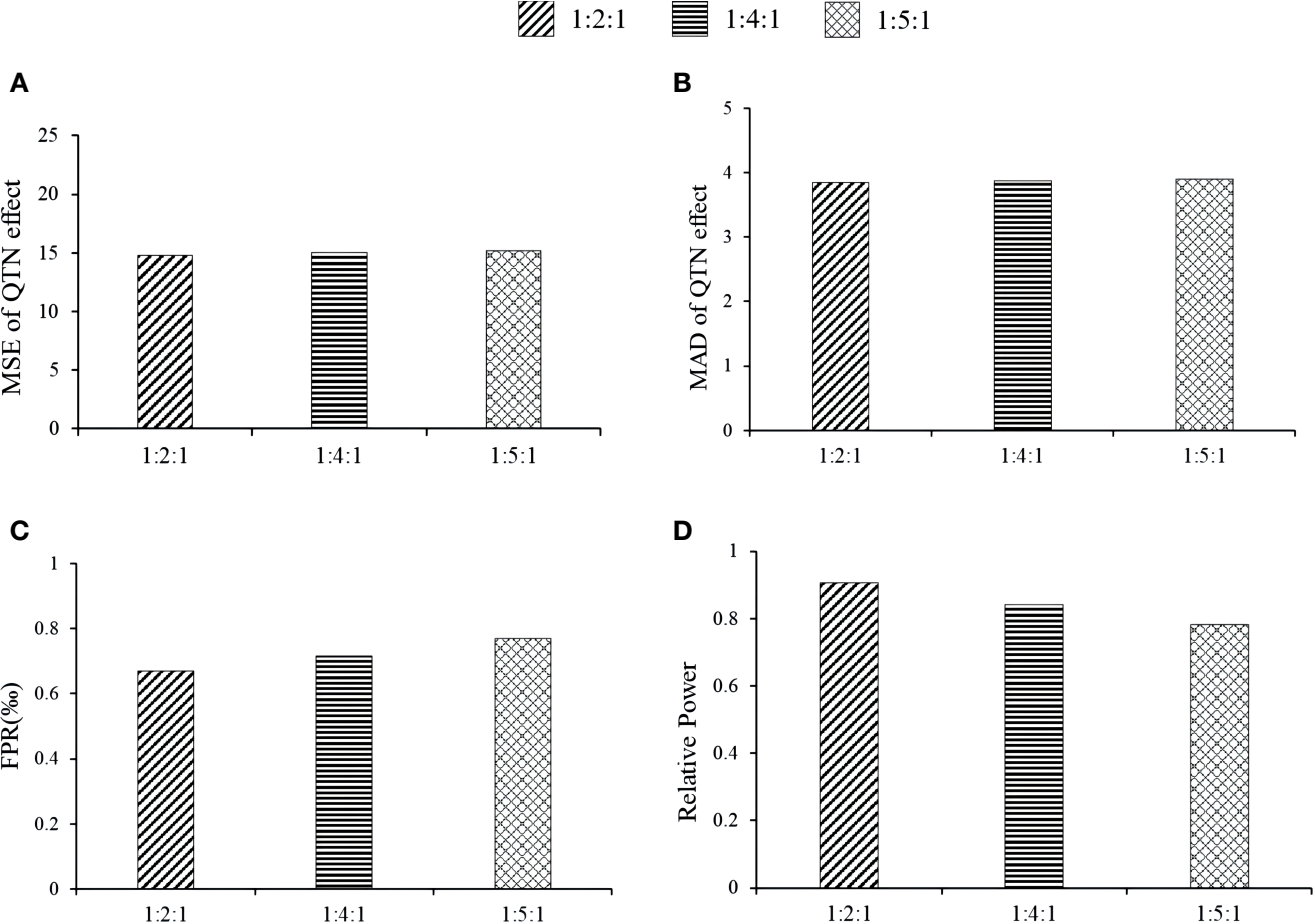
The impact of repetition number of simulation experiment on the association analysis results of CPData (2143 Locus). **(A, B)** MSE and MAD of QTN effect at 2143, respectively; **(C)** false-positive rates; **(D)** relative power.

The fourth simulation study assessed the impact of the number of replicates on the estimates of QTN effects and positions, relative power, and false-positive rate using MTOTC+FASTmrMLM. Based on the results of CPData2 (1:1), CPData3 (1:3:1), and CPData5 (1:2:4:2:1), 10 replicates were set at equal intervals from 1,000 to 10,000. As a result, the results across various numbers of replicates at each locus and for each hierarchical levels (CPData2, CPData3, and CPData5) were insignificant (**Figure 5**). This indicated that the number of replicates did not affect the power, false-positive rate, and the estimates of QTN effects and positions. Therefore, 1,000 replicates were used in subsequent simulation studies.

#### 3.1.5 The effect of distribution proportion skewness on the new method

In the fifth simulation study, we investigated the effect of distribution proportion skewness on the new method under three hierarchical levels. Here the distribution proportion skewness were set as symmetrical distribution (distribution proportion, 1:2:1), uniform distribution (1:1:1), and skewed distribution (4:2:1). The indicators were the relative power, false-positive rate, the estimates of QTN effects and positions. The skewed distribution had the lowest relative power at loci 278, 2143, and 3698, followed by the uniform distribution, and the symmetrical distribution (Supplementary **Figure 2**). The MAD and mean squared error (MSE) of QTN position estimates showed unbiasedness under the three distribution proportion skewness. The skewed distribution (7.09‰) was slightly higher false-positive rate than symmetrical distribution (6.71‰) and uniform distribution (6.82‰). When the kurtosis values of the three distributions for the CPData and the OData were compared, it was found that the steepness of the CPData under 1:2:1 was closer to that of the OData (the kurtosis values for the OData, 1:2:1 CPData, 1:1:1 CPData, and 4:2:1 CPData ranged from 2.163–5.415, 1.963–5.412, 1.958–5.196, and 1.980–3.830, respectively). The CPData under 1:2:1 and 1:1:1 and the OData were relatively close in terms of skewness (the skewness of OData, 1:2:1 CPData, 1:1:1 CPData, and 4:2:1 CPData were in the range of −1.001~0.462, −1.466~0.319, −1.256~0.282, and −0.812~0.777, respectively). The skewness of the CPData under 4:2:1 and the OData differed markedly. Therefore, the accuracy of symmetric distribution via MTOTC+FASTmrMLM was higher than that of uniform distribution and skewed distribution.

#### 3.1.6 The effect of distribution proportion kurtosis on the new method

**Figure 6:**
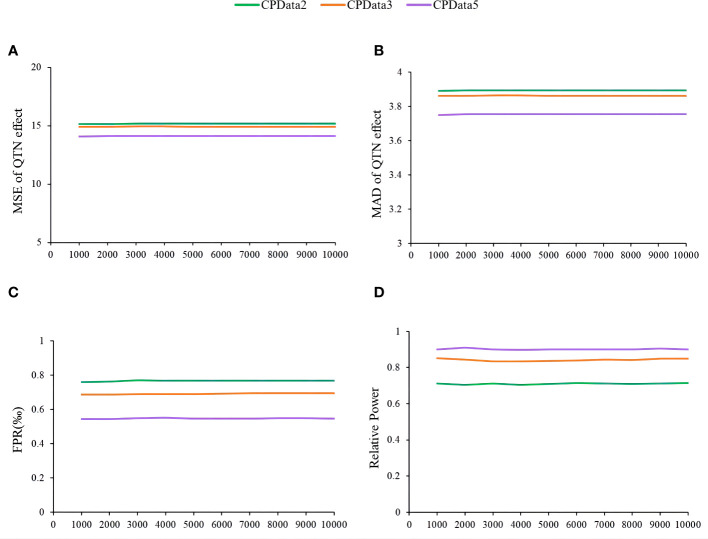
The effect of phenotype distribution kurtosis on the association detection results of MTOTC+FASTmrMLM. **(A, B)** MSE and MAD of QTN effect at 2143, respectively; **(C)** false-positive rates; **(D)** relative power.

Here we studied the effect of distribution proportion kurtosis on the new method. The proportions were set as 1:2:1, 1:4:1, and 1:5:1. The association detection results of the 1:2:1 proportion had the best, e.g., the relative powers of the 1:2:1 proportion at loci 2143, 278, 3698, and 1716 via MTOTC+FASTmrMLM was better than those under others distribution proportion (**Figure 6A**). The MSE and MAD of effect estimates at locus 278, 2143, and 3698 were lower at 1:2:1 than at 1:4:1 and 1:5:1; however, the differences were insignificant (**Figures 6B, C**), while the trends at the other loci were unclear. Under the three distribution proportions, the MSE and MAD of QTN position estimates were all unbiased at loci 278, 2143, 2054, and 3698. However, a lower false-positive rate was observed with the 1:2:1 distribution proportion (**Figure 6D**). Moreover, the steepness of the CPData under distribution proportion 1:2:1 was closer to that of the OData (the kurtosis values of the OData, 1:2:1 CPData, 1:4:1 CPData, and 1:5:1 CPData were 2.163~5.415, 1.963~5.412, 1.967~7.343, and 1.974~7.920, respectively). The skewness showed the same tendency as the kurtosis (the skewness ranges of the OData, 1:2:1 CPData, 1:4:1 CPData, and 1:5:1 CPData were −1.001~0.462, −1.466~0.319, −1.788~0.142, and −1.796~0.150, respectively). In summary, the distribution of the CPData at the 1:2:1 proportion was closer to that of the OData, and MTOTC worked better, compared with the other distribution proportions.

#### 3.1.7 The performance of MTOTC with different GWAS methods

The HData of ordinal trait were transformed by MTOTC, and the obtained CPData were found to be suitable for association analysis via FASTmrMLM when there were five or fewer hierarchical levels, owing to high power. Meanwhile, similar results were obtained when MTOTC was combined with others methods in the mrMLM software (Zhang et al., 2020) (**Supplementary Figure 1**; **Supplementary Table 1**). They were also suitable for GWAS for the CPData of ordinal traits, having the characteristics of high relative power, low false-positive rates, and high accuracy of position and effect estimates. Moreover, similar trends from FASTmrMLM in the simulation experiments with the number of the hierarchical levels and their distribution proportions were observed as well (**Supplementary Figure 2**). MTOTC + FASTmrMLM had the best performance, followed by mrMLM (Wang et al., 2016), ISIS EM-BLASSO (Tamba et al., 2017), and FASTmrEMMA (Wen et al., 2018); and finally by pLARmEB (Zhang et al., 2017) and pKWmEB (Ren et al., 2018). Therefore, MTOTC can be integrated with different methods to conduct GWAS for ordinal traits. Considering the diversity and complexity of phenotypic data in ordinal traits in practice, multiple methods might be simultaneously used in a complementary manner. Accordingly, MTOTC improves the performance in identifying significant loci for ordinal traits.

### 3.2 Real data analysis

To validate the new method, the salt-alkali tolerant data in 286 soybean accessions obtained in 2009 and 2010 from Zhang et al. (2014) was re-analyzed in this study. The experiments were conducted in a completely randomized Design, and the number of high-quality SNP markers in this population was 54,296 (Zhou et al., 2015). First, MTOTC was applied to obtain the CPData. Then, the index data, HData5 [hierarchical data generated from the index data by 1:1:1:1:1 (Shao, 1986)], CPData2 (continuous phenotypic data generated from HData2 by MTOTC), and CPData5 (continuous phenotypic data generated from HData5 by MTOTC) for salt-alkali tolerance in soybean were analyzed using the mrMLM, ISIS EM-BLASSO, pLARmEB, FASTmrEMMA, pKWmEB, and FASTmrMLM methods.

#### 3.2.1 QTNs significantly associated with soybean salt-alkali tolerance

For the four types of phenotypic data of salt-alkali tolerance, a greater number of significant QTNs were detected in CPData than in the index data or HData. Six GWAS methods mapped 65 and 99 QTNs in CPData2 and CPData5 of salt tolerance traits, respectively, and 134 and 60 QTNs in CPData2 and CPData5 of alkali tolerance traits, respectively. pLARmEB detected a greater number of QTNs in CPData (116 for salt tolerance traits and 166 for alkali tolerance traits) compared with the other five GWAS methods, which may be related to its relatively higher false-positive rate. Additionally, the numbers of significant QTNs detected by pKWmEB, mrMLM, and FASTmrMLM in CPData (44, 25, and 14 for the salt tolerance trait and 25, 21, and 19 for the alkali-tolerance trait, respectively) were second only to the number of QTNs detected with pLARmEB.

Four QTNs (locus 9682 on chromosome 2 [Chr2-9682], Chr11-54042, Chr13-64738, and Chr13-65248) for salt tolerance were simultaneously detected in the index data and at least one CPData; however, none of them was detected in HData5. For instance, Chr13-64738 was simultaneously detected in CPData2 by five methods and in the salt tolerance index data by two methods. Chr13-65248 was detected in CPData5 by four methods and in both CPData5 and the index data by FASTmrMLM. Three QTNs (Chr7-34669, Chr13-67342, and Chr20-105040) for alkali tolerance were simultaneously detected in the index data and in at least one CPData, two of them were also detected in HData5.

The results of six GWAS methods for the CPData of salt-alkali tolerance showed that only a few significant QTNs were coincident between 2009 and 2010, which can be explained by the differences in environmental influences between the two years. For salt tolerance, no QTNs were found to overlap between 2009 and 2010 in the six methods. For alkali tolerance, only Chr1-5051 and Chr16-82333 were detected in both years. There was indeed an environmental (year) effect according to variance analysis of the phenotypic results for the two years (Zhang et al., 2014).

#### 3.2.2 Candidate genes for salt-alkali tolerance

Potential candidate genes were mined from 100 kb upstream to 100 kb downstream (Liu et al., 2020) of significant QTNs that were detected in at least two types of data or by two methods (**Tables 2** and **3**). Functional annotation information in the SoyBase database (**Error! Hyperlink reference not valid.** http://www.Soybase.org/) was also used to screen candidate genes. A total of 34 potentially candidate genes for salt tolerance and 25 potentially candidate genes for alkali tolerance were mined.

**Table 2:** Salt stress-related candidate genes from six genome-wide association study methods.

Candidate genes	QTN positions	Methods	Functional annotation	Arabidopsis homologous
*Glyma02g38320*	43804331	mrMLM^1**^, pLARmEB^3**^	transmembrane transport	AT5G22900
*Glyma02g38350*	43804331	mrMLM^1**^, pLARmEB^3**^	Pentatricopeptide repeat (PPR -like) superfamily protein	AT5G37570
*Glyma02g38370*	43804331	mrMLM^1**^, pLARmEB^3**^	zinc ion binding	AT2G40770
*Glyma02g38380*	43804331	mrMLM^1**^, pLARmEB^3**^	catalytic activity	AT5G05200
*Glyma02g38395*	43804331	mrMLM^1**^, pLARmEB^3**^	respiratory burst involved in defense response	AT5G05190
*Glyma04g13670*	13441084	FASTmrEMMA^3**^, mrMLM^3**^, pLARmEB^3**^	oxidoreductase activity	AT4G25240
*Glyma05g25331 ^#^*	31519270	FASTmrEMMA^3*^, ISIS EM-BLASSO^3*^, mrMLM^3*^, pKWmEB^3*^, pLARmEB^3*^	WRKY DNA-binding domain	AT2G34830
*Glyma05g25420 ^#^*	31519270	FASTmrEMMA^3*^, ISIS EM-BLASSO^3*^, mrMLM^3*^, pKWmEB^3*^, pLARmEB^3*^	zinc ion binding	AT5G37930
*Glyma05g25450 ^#^*	31519270	FASTmrEMMA^3*^, ISIS EM-BLASSO^3*^, mrMLM^3*^, pKWmEB^3*^, pLARmEB^3*^	catalytic activity	AT5G44440
*Glyma05g25460 ^#^*	31519270	FASTmrEMMA^3*^, ISIS EM-BLASSO^3*^, mrMLM^3*^, pKWmEB^3*^, pLARmEB^3*^	catalytic activity	AT2G34790
*Glyma08g13260*	9687628	FASTmrEMMA^3**^, FASTmrMLM^3**^, ISIS EM-BLASSO^3**^, mrMLM^3**^, pKWmEB^3**^, pLARmEB^3**^	Serine/threonine protein kinase	AT3G16030
*Glyma10g40400 ^#^*	47864560	FASTmrMLM^2*^, ISIS EM-BLASSO^2*^, mrMLM^2*^, pKWmEB^2*^, pLARmEB^2*^	zinc ion binding	AT5G67450
*Glyma10g40510 ^#^*	47864560	FASTmrMLM^2*^, ISIS EM-BLASSO^2*^, mrMLM^2*^, pKWmEB^2*^, pLARmEB^2*^	zinc ion binding	AT4G15090
*Glyma10g40520 ^#^*	47864560	FASTmrMLM^2*^, ISIS EM-BLASSO^2*^, mrMLM^2*^, pKWmEB^2*^, pLARmEB^2*^	oxidoreductase activity	AT4G33910
*Glyma11g14030 ^#^*	10094063	mrMLM^1**^, pKWmEB^1**^, pLARmEB^3**^	protein serine/threonine kinase activity	AT3G20830
*Glyma11g14040 ^#^*	10094063	mrMLM^1**^, pKWmEB^1**^, pLARmEB^3**^	sequence-specific DNA binding transcription factor activity	AT1G51190
*Glyma11g14050 ^#^*	10094063	mrMLM^1**^, pKWmEB^1**^, pLARmEB^3**^	zinc ion binding	AT1G51200
*Glyma11g14081 ^#^*	10094063	mrMLM^1**^, pKWmEB^1**^, pLARmEB^3**^	catalytic activity	AT3G18080
*Glyma11g14090 ^#^*	10094063	mrMLM^1**^, pKWmEB^1**^, pLARmEB^3**^	transmembrane transport	AT3G20870
*Glyma11g14100 ^#^*	10094063	mrMLM^1**^, pKWmEB^1**^, pLARmEB^3**^	zinc ion binding	AT1G51220
*Glyma11g14110 ^#^*	10094063	mrMLM^1**^, pKWmEB^1**^, pLARmEB^4**^	Zinc finger, C3HC4 type (RING finger)	AT3G63530
*Glyma12g03490*	2356018	FASTmrEMMA^3*^, FASTmrMLM^3*^, ISIS EM-BLASSO^3*^, mrMLM^3*^, pKWmEB^3*^, pLARmEB^3*^	transmembrane transporter	AT2G21050
*Glyma12g03570*	2356018	FASTmrEMMA^3*^, FASTmrMLM^3*^, ISIS EM-BLASSO^3*^, mrMLM^3*^, pKWmEB^3*^, pLARmEB^3*^	catalytic activity	AT4G34980
*Glyma12g03580*	2356018	FASTmrEMMA^3*^, FASTmrMLM^3*^, ISIS EM-BLASSO^3*^, mrMLM^3*^, pKWmEB^3*^, pLARmEB^3*^	transmembrane transporter	AT5G09220
*Glyma13g25266 ^#^*	28469311	FASTmrEMMA^2**^, FASTmrMLM^1,2**^, ISIS EM-BLASSO^2**^, pKWmEB^2**^, pLARmEB^1,2**^	hyperosmotic salinity response	AT1G61120
*Glyma13g27630 ^#^*	30845044	FASTmrEMMA^3**^, FASTmrMLM^1,3**^, mrMLM^3**^, pKWmEB^3**^	protein serine/threonine kinase activity	AT3G20530
*Glyma13g27680 ^#^*	30845044	FASTmrEMMA^3**^, FASTmrMLM^1,3**^, mrMLM^3**^, pKWmEB^3**^	transmembrane transport	AT1G61800
*Glyma13g27691 ^#^*	30845044	FASTmrEMMA^3**^, FASTmrMLM^1,3**^, mrMLM^3**^, pKWmEB^3**^	zinc ion binding	AT4G14220
*Glyma13g27701 ^#^*	30845044	FASTmrEMMA^3**^, FASTmrMLM^1,3**^, mrMLM^3**^, pKWmEB^3**^	response to oxidative stress	AT3G06050
*Glyma13g27710 ^#^*	30845044	FASTmrEMMA^3**^, FASTmrMLM^1,3**^, mrMLM^3**^, pKWmEB^3**^	response to oxidative stress	AT3G06050
*Glyma13g27740 ^#^*	30845044	FASTmrEMMA^3**^, FASTmrMLM^1,3**^, mrMLM^3**^, pKWmEB^3**^	oxidoreductase activity	AT3G06060
*Glyma13g27770 ^#^*	30845044	FASTmrEMMA^3**^, FASTmrMLM^1,3**^, mrMLM^3**^, pKWmEB^3**^	sequence-specific DNA binding transcription factor activity	AT1G54830
*Glyma15g42440*	49869431	FASTmrEMMA^2*^, mrMLM^2*^, ISIS EM-BLASSO^2*^, pKWmEB^2*^, pLARmEB^2*^	Myb-like DNA-binding domain	AT2G44430
*Glyma15g42460*	49869431	FASTmrEMMA^2*^, mrMLM^2*^, ISIS EM-BLASSO^2*^, pKWmEB^2*^, pLARmEB^2*^	Serine/threonine protein kinase	AT2G32850

1: index data; 2: continuous phenotypic data (CPData2) generated from HData2 by MTOTC; 3: continuous phenotypic data (CPData5) generated from HData5 by MTOTC; *: 2009; **: 2010, ^#^: candidate genes were further screened by haplotype block analysis.

**Table 3:** Alkali stress-related candidate genes from six genome-wide association study methods.

Candidate genes	QTN positions	Methods	Functional annotation	Arabidopsis homologous
*Glyma01g41510 ^#^*	53035914	pLARmEB^2,3*^	Protein serine/threonine kinase activity	AT5G60900
*Glyma01g41520 ^#^*	53035914	pLARmEB^2,3*^	sequence-specific DNA binding transcription factor activity	AT4G17500
*Glyma01g41527 ^#^*	53035914	pLARmEB^2,3*^	sequence-specific DNA binding transcription factor activity	AT5G47230
*Glyma01g41560 ^#^*	53035914	pLARmEB^2,3*^	zinc ion binding	AT5G53110
*Glyma01g41581 ^#^*	53035914	pLARmEB^2,3*^	sequence-specific DNA binding transcription factor activity	AT5G47370
*Glyma01g41610 ^#^*	53035914	pLARmEB^2,3*^	sequence-specific DNA binding transcription factor activity	AT3G13540
*Glyma03g28210 ^#^*	36121029	FASTmrEMMA^2**^, pLARmEB^2**^	F-box family protein	AT2G32560
*Glyma03g28222 ^#^*	36121029	FASTmrEMMA^2**^, pLARmEB^2**^	F-box family protein	AT2G26850
*Glyma03g28234 ^#^*	36121029	FASTmrEMMA^2**^, pLARmEB^2**^	F-box family protein	AT2G32560
*Glyma03g28247 ^#^*	36121029	FASTmrEMMA^2**^, pLARmEB^2**^	F-box family protein	AT2G26850
*Glyma07g20380 ^#^*	20580766	FASTmrEMMA^3**^, FASTmrMLM^1,3**^, ISIS EM-BLASSO^3**^, mrMLM^3**^, pKWmEB^3**^, pLARmEB^1,3**^	Pentatricopeptide repeat (PPR) superfamily protein	AT3G48810
*Glyma13g44560 ^#^*	43999096	FASTmrMLM^1*^, pLARmEB^1,3*^, pKWmEB^3*^	transmembrane transport	AT3G19640
*Glyma13g44570 ^#^*	43999096	FASTmrMLM^1*^, pLARmEB^1,3*^, pKWmEB^3*^	sequence-specific DNA binding transcription factor activity	AT4G37850
*Glyma13g44582 ^#^*	43999096	FASTmrMLM^1*^, pLARmEB^1,3*^, pKWmEB^3*^	sequence-specific DNA binding transcription factor activity	AT2G22760
*Glyma13g44594 ^#^*	43999096	FASTmrMLM^1*^, pLARmEB^1,3*^, pKWmEB^3*^	sequence-specific DNA binding transcription factor activity	AT4G37850
*Glyma13g44640 ^#^*	43999096	FASTmrMLM^1*^, pLARmEB^1,3*^, pKWmEB^3*^	Serine/threonine-protein kinase PBS1	AT1G80640
*Glyma13g44660 ^#^*	43999096	FASTmrMLM^1*^, pLARmEB^1,3*^, pKWmEB^3*^	sequence-specific DNA binding transcription factor activity	AT5G25190
*Glyma16g25280 ^#^*	29252235	pLARmEB^2,3*^	sequence-specific DNA binding transcription factor activity	AT2G18350
*Glyma16g25310 ^#^*	29252235	pLARmEB^2,3*^	transmembrane transport	AT1G75220
*Glyma16g25320 ^#^*	29252235	pLARmEB^2,3*^	transmembrane transport	AT1G75220
*Glyma19g39270*	46014852	FASTmrMLM^1*^, pKWmEB^1*^, pLARmEB^1*^	response to oxidative stress	AT4G11290
*Glyma19g39320*	46014852	FASTmrMLM^1*^, pKWmEB^1*^, pLARmEB^1*^	oxidoreductase activity	AT4G03140
*Glyma19g39340*	46014852	FASTmrMLM^1*^, pKWmEB^1*^, pLARmEB^1*^	Regulation of transcription	AT5G62000
*Glyma20g31790 ^#^*	40400845	pLARmEB^1,2*^	zinc ion binding	AT3G52300
*Glyma20g31800 ^#^*	40400845	pLARmEB^1,2*^	transmembrane transport	AT2G35800

1: index data; 2: continuous phenotypic data (CPData2) generated from HData2 by MTOTC; 3: continuous phenotypic data (CPData5) generated from HData5 by MTOTC; *: 2009; **: 2010, ^#^: candidate genes were further screened by haplotype block analysis.

For salt tolerance, 19 candidate genes were detected simultaneously in the index data and CPData5. Among them, *Glyma05g25331*, *Glyma05g25420*, *Glyma05g25450*, and *Glyma05g25460* were all detected by five GWAS methods in CPData5 in 2009. Only one gene, *Glyma13g25266*, was detected in both the index data and CPData2 detected by five GWAS methods in CPData2 and two methods in the index data in 2010. In addition, five candidate genes were detected only in CPData2 by five methods, and nine candidate genes were detected only in CPData5 by three or more methods. No overlapping genes were found between HData5 and the index data or the CPData (**Table 2**).

For alkali tolerance, 7 candidate genes for alkali stress were concurrently detected in the index data and CPData5. For instance, *Glyma07g20380* was simultaneously detected by 2, 1, and 6 GWAS methods in the index data, HData5, and CPData5 in 2010, respectively (**Table 3**). Two candidate genes were detected in the index data and CPData2. Ten candidate genes were simultaneously detected in CPData2 and CPData5. *Glyma10g02920* was detected by one GWAS method in CPData2 and five GWAS methods in CPData5 in 2009. *Glyma07g20380* was detected by all six association analysis methods in CPData5 in 2010.

#### 3.2.3 QTN based haplotype and phenotypic difference analysis

Based on the above 34 salt stress-related candidate genes and 25 alkali stress-related candidate genes, Haploview software was used to perform haplotype block analysis. And the phenotypic differences across haplotypes were examined using the t-test in SAS9.4. Four stable QTNs for salt tolerance and six stable QTNs for alkali resistance were screened to form haplotype blocks based on linkage disequilibrium (**Supplementary Figures 3** and **4**).

In haplotype block with the significant QTNs Chr13-64738 for salt tolerance, t-test showed significant phenotypic differences between haplotypes ACAT and AATT (*P*=0.0341 in 2009 and *P*=0.0083 in 2010), between haplotypes TCAT and AATT (*P*=0.0091) in 2010, and between haplotypes TCAT and TCTT (*P*=0.0471) in 2010. However, for haplotype blocks of other salt tolerance QTNs, it was showed that the significant phenotypic differences existed between haplotypes only in a single year, and the haplotype pairs with significant differences included haplotype AGTGC and TACCC (*P*=0.0348), AGTGC and TGTCA (*P*=0.0345) for Chr5-24153; haplotype GCG and ATA (*P*=0.0408) for Chr10-52140; haplotypes GTAGA and GTAGT (*P*=0.0397), GTAGT and AAGTT (*P*=0.0540) for Chr11-54042.

There were two significant QTNs Chr16-82333 and Chr3-14262 for alkali tolerance with significant phenotypic differences across haplotypes in both years. The Chr16-82333 recorded significant differences between haplotypes CTGACG and CCGGAG (*P*=0.0158 in 2009, *P*=0.0614 in 2010), between haplotypes CTGACG and CCGGAG (*P*=0.0005 in 2009), between haplotypes CTGACG and CCGAAG (*P*=0.0231 in 2009), between haplotypes TCGAAG and CCGAAG (*P*=0.0619 in 2009, *P*=0.0261 in 2010), and between haplotypes CCAAAG and CCGGAG (*P*=0.0296 in 2010). For Chr3-14262, the haplotype pairs with significant differences were detected as follows: TTT and TCT (*P*=0.0217 in 2009, *P*=0.0085 in 2010), TTT and GCT (*P*=0.0102 in 2010), GCT and TCT (*P*=0.0171). The other haplotype blocks of alkali tolerance showed significant phenotypic differences between haplotypes only in a single year and they include: GTGT and TTAT (*P*<0.0001), TTGT and TTAC (*P*=0.0038), TTAT and TAGT (*P*=0.0132) for Chr13-67342; CAG and TGT (*P*=0.0183) for Chr1-5051; ATCG and GATC (*P*=0.0009) for Chr7-34669; TAGGCG and AATGCA (*P*=0.0157), and TAGGCG and TATGCG (*P*=0.0128) for Chr20-105040.

Genes with significant phenotypic differences across haplotypes were considered as the candidate genes (**Tables 2** and **3**), including 22 salt stress-related candidate genes and 22 alkali stress-related candidate genes. Among them, six salt stress-related candidate genes (*Glyma05g25420*, *Glyma11g14030*, *Glyma11g14040*, *Glyma11g14050*, *Glyma13g27691*, *Glyma13g27701*) and six alkali stress-related candidate genes (*Glyma03g28222*, *Glyma03g28234*, *Glyma03g28247*, *Glyma16g25320*, *Glyma20g31790*, *Glyma20g31800*) were found in the haplotype block.

## 4 Discussion

In this study, we established a method for transforming ordinal phenotypes into continuous phenotypes (MTOTC) based on hierarchical data for ordinal trait phenotypes and molecular marker data in resource populations. Therefore, the process of association analysis for ordinal traits is as follows: first, MTOTC is used to transform HData into continuous phenotypic data (CPData), and then a C-GWAS method (i.e. GWAS method for continuous quantitative traits) is selected to analyze the CPData to identify the QTNs that are significantly associated with ordinal traits.

In this study, simulation experiments and soybean saline-alkali tolerance analysis indicated that the new method, MTOTC, is suitable for ordinal traits when they are less than five hierarchical levels. Moreover, the combination of MTOTC with any one of the proposed C-GWAs methods exhibited high power, low false-positive rates, and low bias in estimating the positions and effects of the QTN. The purpose of MTOTC is to provide a different approach for undertaking GWAS for ordinal traits. The feasibility of the MTOTC method was verified in real data analysis of soybean salt-alkaline tolerance using 286 soybean accessions. Compared with HData5 (i.e., the data classified as five hierarchical levels), a greater number of significant QTNs was detected concurrently by at least two GWAS methods or in two years, and more candidate genes for salt and alkali stress were screened in the CPData for salt and alkali tolerance traits. A greater number of QTNs was detected simultaneously by multiple GWAS methods in the CPData than in the index data and HData for salt-alkaline tolerance. For the three types of data, the number of QTNs detected simultaneously was respectively 4, 1, and 1 in salt tolerance and respectively 5, 2, and 3 in alkali resistance.When the phenotype distribution of the CPData generated by the new method were closer to those from the index data of salt-alkali tolerance, the GWAS results were better, and a greater number of candidate genes could be mined. This may be beneficial for selecting the appropriate distribution proportion to obtain hierarchical data of ordinal trait, screening stable QTNs, and promoting the development of molecular breeding. We also applied symmetric distribution (1:2:4:2:1) to generate HData5 for the salt tolerance index data and used MTOTC to generate the corresponding CPData5. The phenotype distribution of CPData5 with symmetric 1:2:4:2:1 exhibited a large deviation from that of the index data, and the phenotype distribution of CPData5 with uniform 1:1:1:1:1 was closer to that of the index data. Under the six methods, there were no overlapping QTNs in CPData5 and the index data for salt tolerance, which was far inferior to the above uniform distribution observed with the distribution proportion 1:1:1:1:1, under which three coincident QTNs were detected in CPData5 and the index data. This result corresponded precisely to the results presented in simulation study 5.

MTOTC performed well in the initial SNP screening. After preliminary screening under a *P ≤* 0.05 threshold, a large number of SNPs that were significantly unrelated to the trait could be eliminated. Meanwhile, the simulation experiment showed that the retention rates of related loci remained high. MTOTC serves to simplify the model and save a substantial amount of computing time for subsequent association studies.

MTOTC helps to improve association analyses of ordinal traits. Regarding coefficient of variation, skewness, kurtosis, and frequency distribution, compared with the HData, the results obtained for the CPData were closer to those of the OData. Meanwhile, the results using six GWAS methods showed that the statistical power, the false-positive rate, and the position estimates in CPData were better than those in HData. Moreover, MTOTC performed better when the frequency distribution of the CPData was close to that of the OData.

The fewer hierarchical levels, the more suitable MTOTC is. Regarding the relative power in CPData under different hierarchical levels, a trend of increasing relative power with increasing number of hierarchical levels was found for all six methods when there were four or less hierarchical levels. When there were five hierarchical levels, the power of MTOTC+FASTmrMLM was close to that of FASTmrMLM in HData, but slightly lower than the power from logistic regression; only three GWAS methods had higher relative power in CPData than in HData. In addition, MTOTC had a tendency to increase variation, especially with increasing numbers of hierarchical levels. This indicates that MTOTC is more suitable for ordinal traits with fewer hierarchical levels, especially those with two or three levels. Among the six GWAS methods, FASTmrEMMA, FASTmrMLM, and mrMLM are significantly better when combined with MTOTC. This is partly attributed to that the distribution and parameter estimation principles set in MTOTC were relatively consistent with those in these three GWAS models.

This study will contribute to further research in association analysis of ordinal traits. This is especially in improving the retention rate of small-effect loci in preliminary screening, reducing the impact on variability when transforming ordinal phenotypes into continuous phenotypes, and developing novel methods for association analyses of ordinal traits.

## Data availability statement

The original contributions presented in the study are included in the article/**Supplementary Material**. Further inquiries can be directed to the corresponding author.

## Author contributions

MY, JF, and YW designed the methodologies. MY, JF, JZ, and JCZ drafted the manuscript, conducted simulation studies, and analyzed the data. TZ and JF revised the paper. All authors contributed to the article and approved the submitted version.
