# Supplementary material for: Improving power of genome-wide association studies via transforming ordinal phenotypes into continuous phenotypes
Source: Front Plant Sci. 2023 Nov 2;14:1247181. doi: 10.3389/fpls.2023.1247181 (PMC10652869; doi:10.3389/fpls.2023.1247181)
Supplement: Supplementary file 1 [file DataSheet_1.docx]

Supplementary Material

Improving power of genome-wide association studies via transforming ordinal phenotype into continuous phenotypes

Ming Yang^1†^, Yangjun Wen^2†^, Jinchang Zheng^1^, Jin Zhang^2^, Tuanjie Zhao^1^, Jianying Feng^1*^

*** Correspondence:**

Jianying Feng:

[fengjianying@njau.edu.cn](mailto:fengjianying@njau.edu.cn)


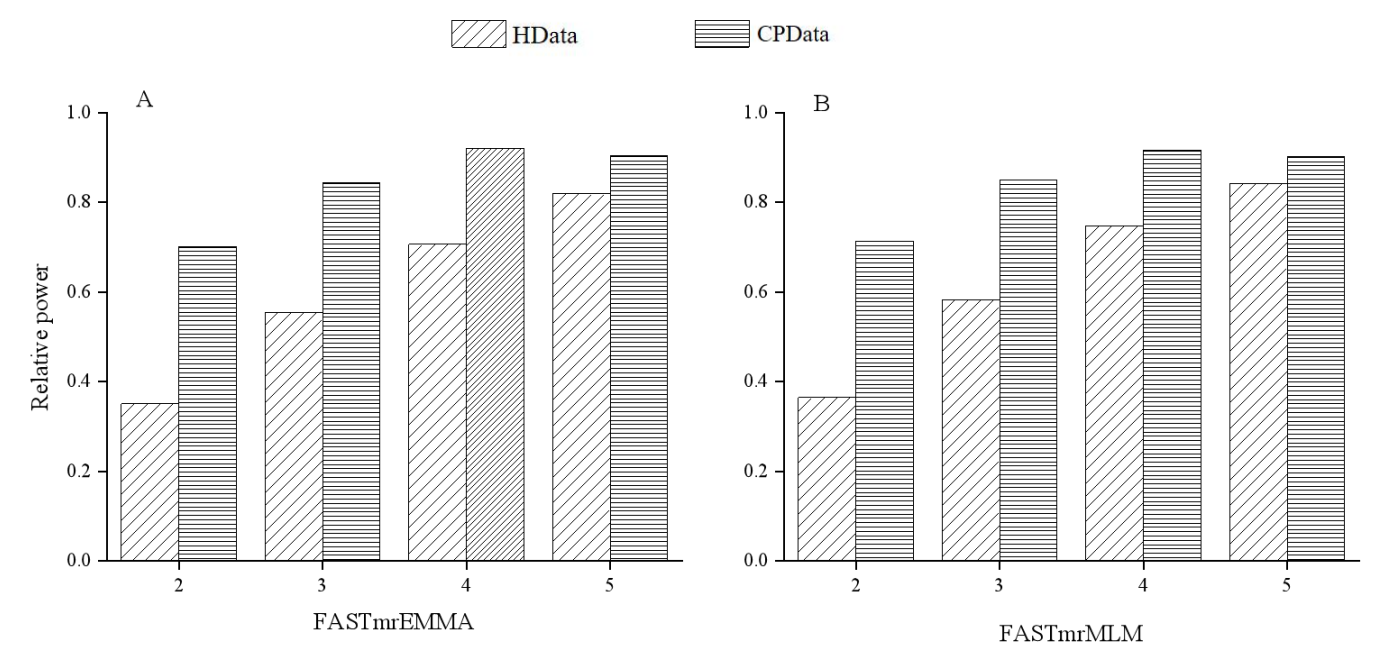

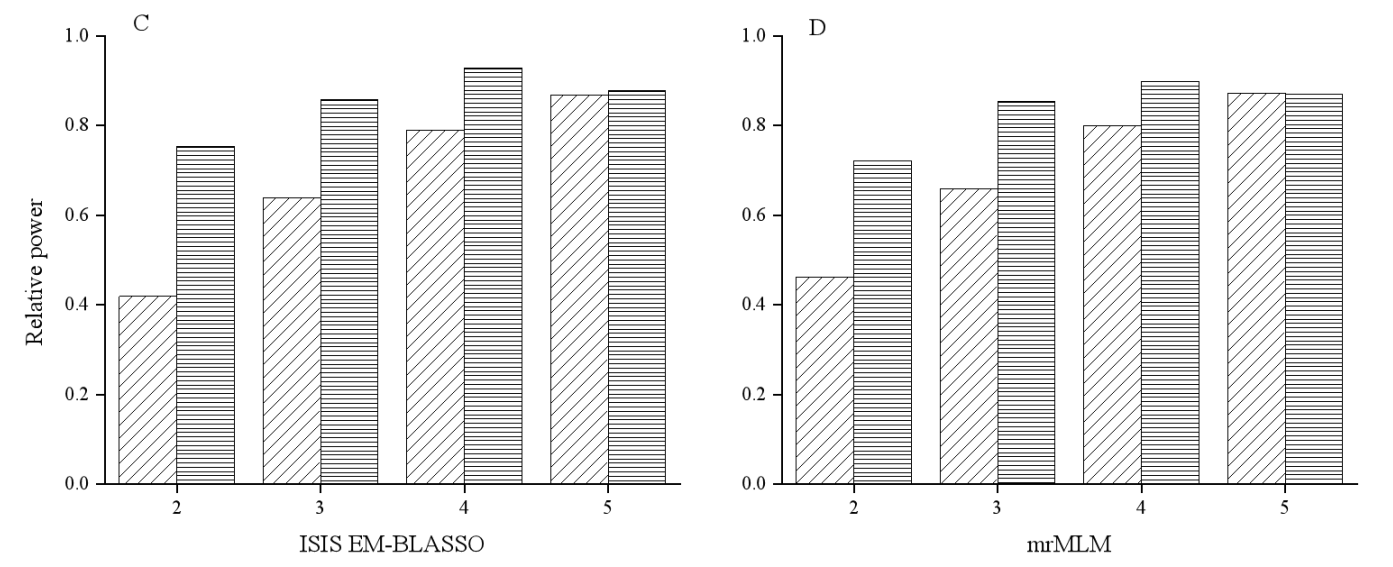

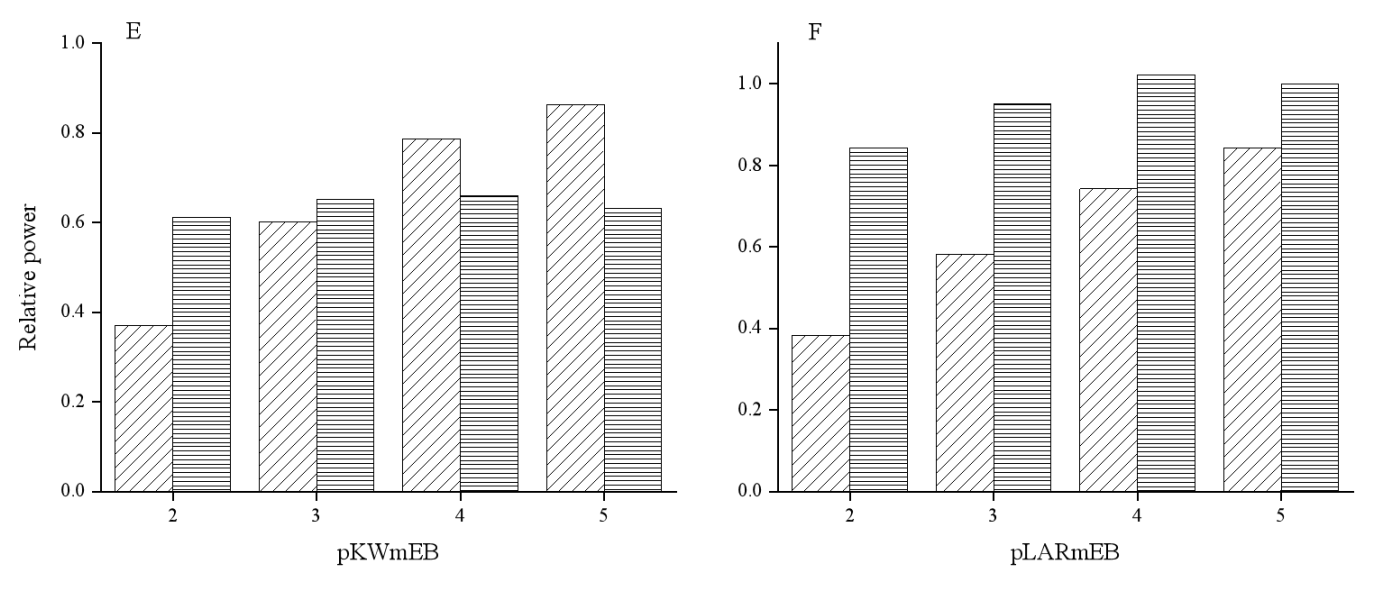


**Supplementary Figure 1.** The relative power of six GWAS methods for HData and CPData under the different number of hierarchical levels (2143 Locus)

**Supplementary Figure 2.** Relative power of six association analysis methods for CPData in three types of distribution proportion


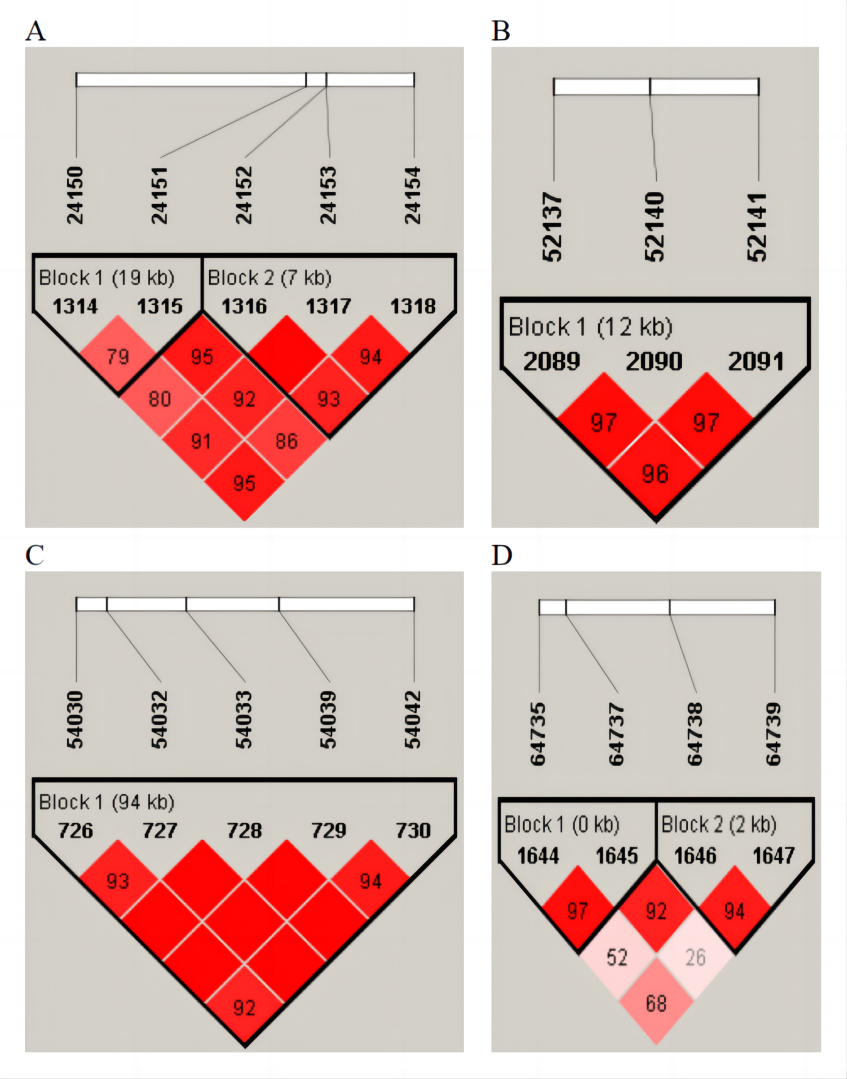


**Supplementary Figure 3.** Haplotype block analysis at the stable QTNs related to salt tolerance


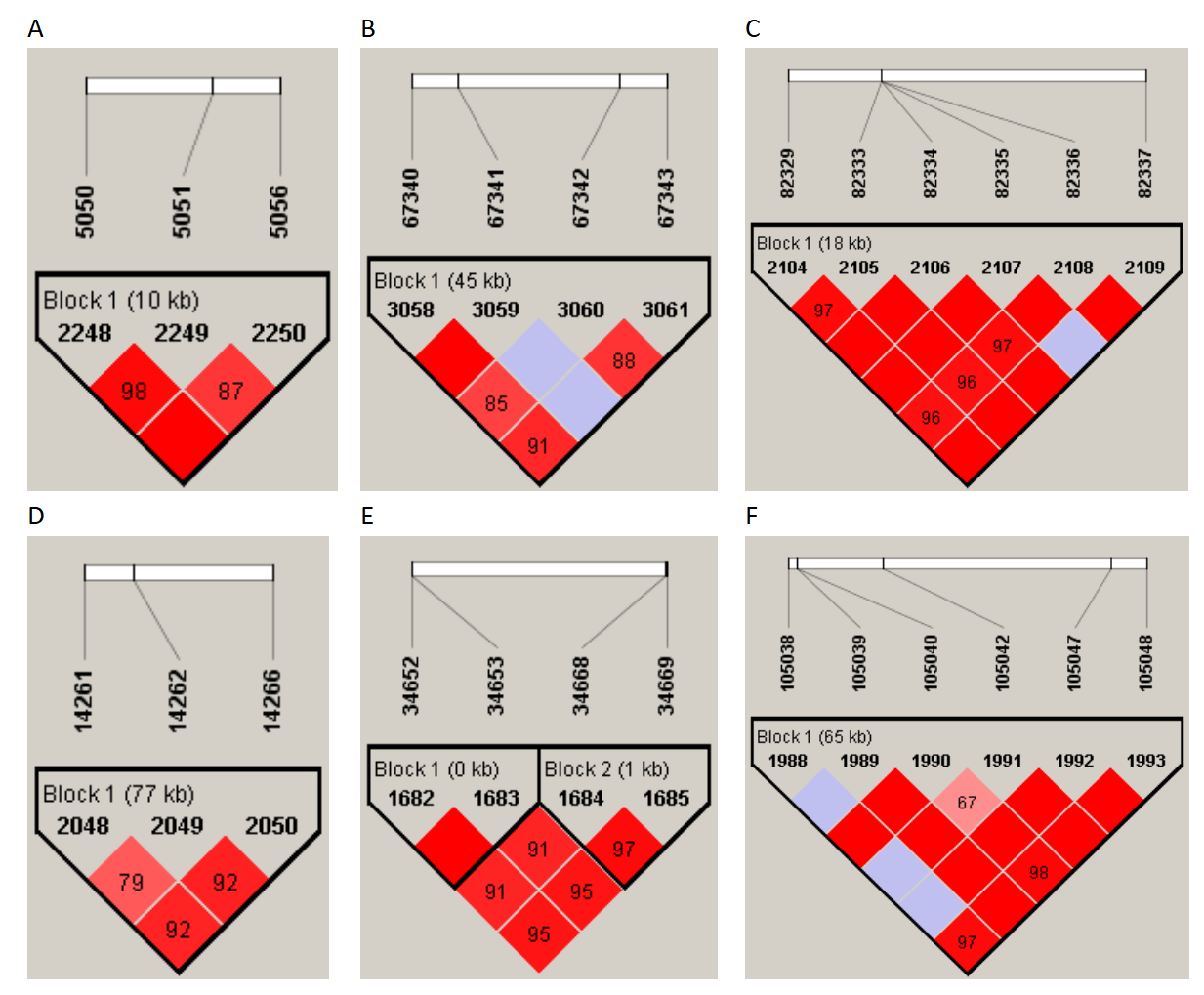


**Supplementary Figure 4.** Haplotype block analysis at the stable QTNs related to alkali tolerance

**Supplementary Table 1.** MSE and MAD of QTN position in OData, HData and CPData under six GWAS methods (*N*=2)

| Methods | Chr | Loci | h^2^ | Pos | MSE of QTN position | | | MAD of QTN position | | |
| --- | --- | --- | --- | --- | --- | --- | --- | --- | --- | --- |
|  |  |  |  |  | OData | HData2 | PCData2 | OData | HData2 | PCData2 |
| FASTmrEMMA | 1 | 278 | 0.1 | 11298364 | 3853.281553 | 9057.477623 | 0 | 14.12427184 | 33.29955008 | 0 |
|  | 2 | 2143 | 0.15 | 5134228 | 4.285186705 | 18.99961014 | 0 | 0.072630283 | 0.32202729 | 0 |
|  | 2 | 3698 | 0.05 | 6119482 | 0 | 0 | 0 | 0 | 0 | 0 |
| FASTmrMLM | 1 | 278 | 0.1 | 11298364 | 3201.380179 | 7460.274313 | 0 | 11.7276472 | 27.42747909 | 0 |
|  | 2 | 2143 | 0.15 | 5134228 | 6.654983431 | 19.46051712 | 0 | 0.112796329 | 0.329839273 | 0 |
|  | 2 | 3698 | 0.05 | 6119482 | 0 | 0 | 0 | 0 | 0 | 0 |
| ISIS EM-BLASSO | 1 | 278 | 0.1 | 11298364 | 3263.120656 | 8033.919596 | 0 | 11.99676712 | 29.50620922 | 0 |
|  | 2 | 2143 | 0.15 | 5134228 | 7.006511958 | 28.50056465 | 0 | 0.11875444 | 0.483060418 | 0 |
|  | 2 | 3698 | 0.05 | 6119482 | 0 | 0 | 0 | 0 | 0 | 0 |
| mrMLM | 1 | 278 | 0.1 | 11298364 | 8342.670632 | 12569.20283 | 0 | 30.6715832 | 46.21030454 | 0 |
|  | 2 | 2143 | 0.15 | 5134228 | 1.524525547 | 14.2934302 | 0 | 0.025839416 | 0.242261529 | 0 |
|  | 2 | 3698 | 0.05 | 6119482 | 0 | 0 | 0 | 0 | 0 | 0 |
| pKWmEB | 1 | 278 | 0.1 | 11298364 | 3999.135135 | 8256.337286 | 0 | 14.7027027 | 30.32712215 | 0 |
|  | 2 | 2143 | 0.15 | 5134228 | 7.608743169 | 20.53014417 | 0 | 0.128961749 | 0.347968545 | 0 |
|  | 2 | 3698 | 0.05 | 6119482 | 0 | 0 | 0 | 0 | 0 | 0 |
| pLARmEB | 1 | 278 | 0.1 | 11298364 | 4899.199214 | 8222.257111 | 0 | 17.96904937 | 30.22888644 | 0 |
|  | 2 | 2143 | 0.15 | 5134228 | 9.867542842 | 55.10508589 | 0 | 0.167246489 | 0.933984507 | 0 |
|  | 2 | 3698 | 0.05 | 6119482 | 0 | 0 | 0 | 0 | 0 | 0 |

*N*: the number of hierarchical levels.
